# Factors Influencing Escalator-Related Incidents in China: A Systematic Analysis Using ISM-DEMATEL Method

**DOI:** 10.3390/ijerph16142478

**Published:** 2019-07-11

**Authors:** Kefan Xie, Zimei Liu

**Affiliations:** School of Management, Wuhan University of Technology, Wuhan 430070, China

**Keywords:** escalator incidents (EIs), influencing factors, ISM-DEMATEL method, hierarchical structure model, preventive measures, injury prevention

## Abstract

Escalator-related incidents (EIs) have recently resulted in serious injuries and even deaths. Given the frequency and severity of EIs, a systematic exploration of factors influencing EIs is critical in order to identify preventive measures. Twenty-two factors influencing EIs were identified by analyzing 213 EI cases in China and related literatures. A combination of the Interpretive Structural Modeling (ISM) and Decision Making Trial and Evaluation Laboratory (DEMATEL) methods were utilized to establish a hierarchical structure of the influencing factors and to distinguish cause factors and effect factors. The results show: (i) behavior, emergency plan, safety rules, safety supervision, information exchange, safety culture, and safety education are the most important factors influencing EIs; (ii) safety education is the cause factor imposing the greatest influence on other factors while behavior is the effect factor that is the most influenced; and (iii) the structure of influencing factors has five hierarchies, and factors in the root cause layer are settings and components, safety rules, safety supervision, safety culture, and safety education. Management priority should be given according to the hierarchy level, and the interaction of factors should be considered when taking preventive measures. The corresponding five-layer countermeasures are proposed to reduce escalator-related injuries.

## 1. Introduction

An escalator is a type of vertical transportation in the form of moving staircases. Along with common transportation instruments like subways and trains, escalators are a special and important part of the public transportation system that enable people to travel to their destinations. However, the dynamic interaction of escalator-to-passenger, passenger-to-passenger, passenger-to-environment, and passenger-to-management forms a complex escalator–passenger–environment–management (EPEM) system. Safety risks in this system are unpredictable, hard to identify and difficult to control, increasing the likelihood of EIs. The recent emergence of EIs in various countries has brought many negative effects on public safety and social sustainability. On 23 October 2018, an escalator incident in a subway station in Rome, Italy injured more than 20 passengers. According to General Administration of Quality Supervision Inspection and Quarantine (AQSIQ) statistics, 56 serious EIs occurred in China in 2017 and 31 occurred in 2018. EIs have consistently been the leading cause of injuries and deaths related to special equipment from 2008 to 2018 in China. A number of serious EIs were reported by mainstream media in recent years and are listed in Table 1. Preventing the occurrence of EIs has become an issue worthy of attention.

Statistical studies on EIs were conducted to analyze the frequency distribution of factors such as cause, hazard pattern, injured body part, gender, and age. Xing et al. identified the main causes of EIs in subway stations in Guangzhou, China as failing to keep balance, carrying out other tasks, not holding the handrail and unhealthy passengers [1]. O’Neil et al. found that escalator-related injuries among old adults in the U.S. were mainly caused by a slip, trip, or fall [2]. Chi et al. investigated 194 EIs cases and suggested that strict design code and in-depth incident investigation are effective measures to prevent incidents [3]. McGeehan et al. studied 26,000 escalator-related injuries of children in the U.S. and found that falls and entrapments were the crucial causes of injuries [4]. Additionally, some studies examining EIs were either case studies or focused on children and old adults [5,6,7].

Simulation methods were utilized to study the evolution process of EIs in a virtual world [8]. Li et al. used simulation tools to study the impact of key factors on group stampede risk during escalator transfer [9]. They concluded that the propagation speed of the incident was always faster than the recovery rate, and timely taking emergency measures can reduce the severity of the accident. Li et al. analyzed the impact of contributing factors on individual stampede probability, and the impact of four factors were queued in descend order: pedestrian traffic, picking-up duration, pedestrian velocity, and escalator velocity [10]. Kauffmann and Kikuchi calculated the practical capacity of escalators through simulation based on passenger behavioral rules and found that prohibiting walking on the escalator can improve evacuation efficiency in emergency scenarios by reducing variability in the escalator system [11].

In the EPEM system, however, EIs are much more likely to be caused by multiple influencing factors rather than one single factor. The dynamic interaction of factors usually constitutes multiple influencing loops to form a complex structure. Influencing factors of EIs have not been adequately analyzed through a traditional linear analysis or simulation method mentioned above. Further in-depth understanding of the influencing mechanism of the factors is crucial for EPEM system safety.

ISM is an analytical method exploring complex socio-economic system problems. It is also an important structural modeling technique for system analysis [12,13,14]. DEMATEL is an approach using graph and matrix theory to analyze system factors [15]. ISM emphasizes establishing hierarchical structure models of complicated systems according to relationships of system factors, while DEMATEL focuses on identifying key factors of systems and distinguishing cause and effect factors. The integrated ISM-DEMETAL method has been widely used to overcome the weaknesses of applying either alone as they are complementary in function. Wang et al. defined the factors of mining safety and revealed the influencing mechanisms by using ISM-DEMETAL method [16]. Fu et al. clarified system structure of communication networks through ISM-DEMETAL and entropy [17]. Shen et al. constructed the multi-level hierarchical structure of factors affecting distributed natural gas-combined cooling, heating, and power systems in China [18]. Mousavizade and Shakibazad studied the critical success factors of knowledge management in Iranian urban water and sewage companies [19]. In this paper, the ISM-DEMETAL method was adopted to analyze the influencing factors of EIs extracted from 213 EIs cases in China and related literatures.

This paper systematically analyzed influencing factors of EIs in China and accordingly proposed preventive measures, providing theoretical support for safety management of escalator passengers. The main aims of this paper were: (a) to identify the influencing factors of EIs and distinguish the importance and degree of influence of each factor; (b) to establish the hierarchical structure of the factors and analyze the influencing mechanism; and (c) to correspondingly propose preventive measures to reduce escalator-related injuries.

## 2. Materials

### 2.1. Data Resource

News is an original record with authoritative description, which provides the latest statistics and the detailed information [20]. A Web crawler tool was adopted to obtain Chinese news reports of EIs published between September 2003 and February 2019. Data was recorded from news sites including China News, China Daily, Xinhua Net, People Net, Baidu News, Sina News, CNTV and Youth News [21]. “Escalator injury”, “escalator incident” and “escalator death” were used as subject terms to conduct the retrieval of related news. After dealing with duplicate data, a total of 213 cases of EIs were collected.

Thirty keywords which represent the core topic of EI cases, occurring at least 10 times, were chosen as samples for the descriptive analysis, as listed in Table 2. These keywords exemplify some traits of EIs in China. Firstly, the most common places of EIs are “escalator” (417) in “subway station” (79), “shopping mall” (62), “supermarket” (47), and “plaza” (35). Secondly, “child” (249), “elder” (161), and “women” (54) are the main victims of EIs. Thirdly, the most common hazard pattern of EIs in China are “stumble” (229), “entrap” (67), “stuck” (45), “roll” (40), “slip” (24), “draw” (19), and “fall” (11). Fourthly, keywords such as “injure” (130), “medical care” (90), “fracture” (15), “death” (11), and “graze” (11) reflect that the EIs usually lead to serious consequences for passengers. Fifthly, “handrail” (68), “gap” (35), and “emergency button” (33) are the most frequently mentioned escalator mechanical structures in the incident reports. Finally, the most commonly injured body parts of passengers are “hand” (41), “foot” (35), “finger” (27), “arm” (17), and “head” (11).

### 2.2. Influencing Factors

According to the analysis of 213 cases in China, each EI was the result of multiple factors, not a single factor. The multiple factors make the EPEM system uncertain and opaque overall, reducing the ability of managers to reduce risk and exacerbating the consequences [22]. Based on collected cases and related literatures, influencing factors of EIs were identified and described in four main areas, i.e., passenger, escalator, environment, and management.

#### 2.2.1. Passenger Factors

Basir et al. conducted research on escalator incidents and found that the main factors in contributing to incidents were passenger factors which are dynamic and complicated [23]. Traditional linear risk model is insufficient to explore the impact of passenger factors on incidents [24]. In this paper passenger factors were analyzed in two dimensions: passenger characteristic and passenger behavior. Passenger characteristic reflects reaction capacity and action capability of the passenger, which affect the safety level of the EPEM system. Passenger characteristic includes physical state such as age and gender, and other features such as mental condition, clothing and safety education level. Escalator-related injuries are believed to be closely related to passenger age, gender and mental condition [1]. Passenger behavior impacts public transportation safety, which has been widely discussed in previous studies [25,26,27,28,29]. Passenger factors were comprehensively considered from eight perspectives: age and gender, mental or physical condition, outfits and belongings, behavior, knowledge about escalators, safety awareness, safety cognition, and safety skill.

#### 2.2.2. Escalator Factors

Escalator factors refer to the factors related to the operation of escalators which might have encouraged, facilitated, or enabled the incident to happen, including type, size, materials, service term, labeling, safety devices, malfunctions and maintenance. Al-Sharif et al. found that escalator braking should be set at 1.16 m·s^−2^ in order to eliminate the risk of passenger falls caused by unplanned stoppages [30]. Escalator factors were analyzed from four aspects including settings and components, operating status, safety devices, and maintenance.

#### 2.2.3. Environmental Factors

Environmental factors are the factors related to the surroundings of escalators which might have contributed to EIs. The operating environment of escalator is rather complex and has many aspects, such as location, surrounding object, ground, lighting and visibility, and signage. By analyzing previous research results, installation location, size of space, signage, and physical environment were selected as the main environmental factors to be analyzed.

#### 2.2.4. Management Factors

When implementing complex system safety control, human factors, management factors, and organizational factors should be fully considered. Organizational management, safety culture, and employee participation are important to prevent safety incidents in companies [31]. Management errors repeatedly appear in safety incidents. Komljenovic et al. found that analyzing management weakness is useful for probing underlying factors of safety incidents [32]. These studies suggest the importance of management factors in incident prevention. Safety performance has a close relationship with safety culture [33]. Nordlöf et al. concluded that safety culture is closely related to occupational health and safety management based on the statistical analysis [34]. This paper analyzed the effect of safety management from six aspects including safety rules, safety supervision, information exchange, safety culture, emergency plan, and safety education. Twenty-two influencing factors of EIs are summarized and listed in Table 3.

## 3. Method

**Figure 1:**
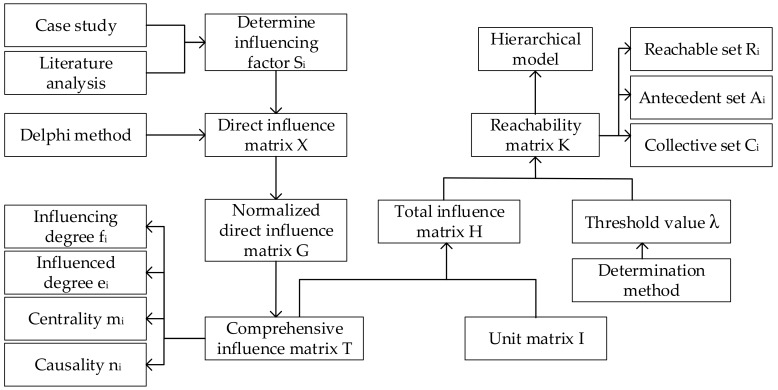
The framework of Interpretive Structural Modeling and Decision Making Trial and Evaluation Laboratory (ISM-DEMATEL) method.

ISM-DEMATEL is a systems analysis approach utilizing matrix and graph theory to analyze the relationship between system factors. The combination of ISM and DEMATEL can not only identify the influence degree and importance of factors in the EPEM system, but also comprehensively analyze the hierarchical structure of the system. The framework of the ISM-DEMATEL method is illustrated in Figure 1.

The Equations (1)–(12) for calculating the matrices are listed below. The detailed steps of ISM-DEMATEL method can be found from the related literatures [16,18].

(1) $$X=\left[\begin{array}{cccc} 0 & x_{12} & \cdots & x_{1n}\\ x_{21} & 0 & \cdots & x_{2n}\\ \vdots & \vdots & \ddots & \vdots \\ x_{n1} & x_{n2} & \cdots & 0 \end{array}\right]$$

(2) $$G=\frac{1}{\underset{1\leq i\leq n}{\max}\sum_{j=1}^{n}x_{ij}}X$$

(3) $$T=G{\left(I-G\right)}^{-1}$$

(4) $$\;f_{i}=\overset{n}{\underset{j=1}{\sum }}t_{ij}\left(i=1,2,\ldots ,n\right)$$

(5) $$\;e_{i}=\overset{n}{\underset{j=1}{\sum }}t_{ji}\left(i=1,2,\ldots ,n\right)$$

(6) $$m_{i}=\;f_{i}+\;e_{i}\left(i=1,2,\ldots ,n\right)$$

(7) $$n_{i}=\;f_{i}-\;e_{i}\left(i=1,2,\ldots ,n\right)$$

(8) H=T+I

(9) $$k_{ij}=\left\{\begin{array}{c} 1,h_{ij}\geq \lambda ,\;i=1,2,\ldots ,n\;\\ 0,h_{ij}<\lambda ,\;i=1,2,\ldots ,n \end{array}\right.$$

(10) $${\;R}_{i}=\left\{\;S_{j}|\;S_{j}\in X,k_{ij}=1\;\right\},\left(i=1,2,\ldots ,n\right)$$

(11) $$A_{i}=\left\{\;S_{j}|\;S_{j}\in X,k_{ji}=1\;\right\},\left(i=1,2,\ldots ,n\right)$$

(12) $$C_{i}={\;R}_{i}\cap A_{i}$$

## 4. Results

### 4.1. Establishment of Direct Influence Matrix

Based on case study and literature analysis, 22 factors influencing EIs, denoted as $S_{1},{\;S}_{2},{\;S}_{3},\;\ldots ,{\;S}_{n}$ were identified in Section 2.

Influence relationships between these factors are determined through Delphi method. A 3-point Likert-type scale was designed. Twenty questionnaires were collected from safety management experts in universities, safety managers in subway stations and escalator manufacturers in Wuhan. Questionnaire data were averaged, and the average values were rounded-off to determine the measure of the direct relationship between every two factors. The direct influence matrix X was obtained and is shown in Table S1 in Supplementary Materials. $x_{ij}=0$ indicates that factor $S_{i}$ has no influence on factor $S_{j}$. $x_{ij}=1$ suggests that $S_{i}$ has a weak influence on $S_{j}$ and $x_{ij}=2$ means that $S_{i}$ has a strong influence on $S_{j}$. $x_{ij}=x_{ji}=0$ when i=j.

### 4.2. Establishment of Comprehensive Influence Matrix

A direct influence matrix reflects the direct relationship between influencing factors. However, considering just the direct relationship is inadequate, since multiple factors affect the safety risk in the EPEM system. A comprehensive influence matrix reflects the comprehensive relationship among system factors, including both direct and indirect relations. The comprehensive influence matrix T was calculated and is reported in Table S2.

### 4.3. Calculation of Influencing Degree, Influenced Degree, Centrality, and Causality

Based on the comprehensive influence matrix, influencing degree, influenced degree, centrality and causality of each factor were calculated via Equations (4)–(7). The results are listed in Table 4.

**Figure 2:**
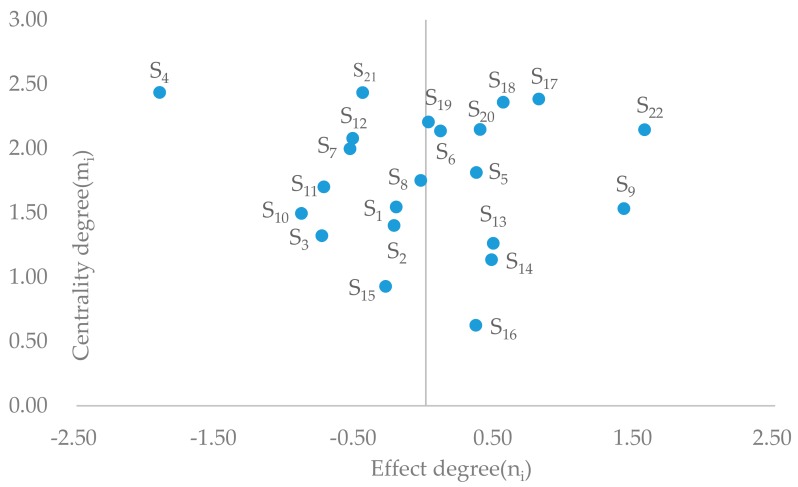
The cause-and-effect relation diagram.

The cause-and-effect relation diagram of the influencing factors is drawn using $n_{i}$ as the *x*-axis and $m_{i}$ as the *y*-axis, as shown in Figure 2.

The influencing degree and influenced degree of the factors clearly seen from Table 4. Eight factors have large influencing degrees, including safety education S_22_, safety rules S_17_, settings and components S_9_, safety supervision S_18_, safety culture S_20_, safety awareness S_6_, information exchange S_19_, and knowledge about escalators S_5_. These factors are not clearly visible but are very important, as they can affect escalator passenger safety by influencing other factors. Actions should be taken with respect to these factors so as to make a positive change in other factors and eventually lead to a higher safety level of the EPEM system. In the influenced degree ranking, behavior S_4_, emergency plan S_21_, maintenance S_12_, safety cognition S_7_, safety devices S_11_, and operating status S_10_ are the top six factors, indicating that enhancing their influence has a direct impact on improving passenger safety.

Centrality $m_{i}$ reflects the position and importance of the factor $S_{i}$ in the system. Behavior S_4_ is the most important factor, followed by emergency plan S_21_, safety rules S_17_, safety supervision S_18_, information exchange S_19_, safety culture S_20_, safety education S_22_, and safety awareness S_6_. The centrality value of these factors is greater than 2.10 in Table 4, and these factors occupy the top region of Figure 2. Two of them are passenger factors and the remaining four are management factors, suggesting that strengthening safety management and enhancing safety awareness are prime means of controlling EI risk. 

The causality value of the cause factors is greater than 0, and these factors are on the right region of Figure 2. Half of the 22 factors are cause factors, including safety education S_2_, settings and components S_9_, safety rules S_17_, safety supervision S_18_, installation location S_13_, size of space S_14_, safety culture S_20_, knowledge about escalators S_5_, physical environment S_16_, safety awareness S_6_, and information exchange S_19_, while the rest are effect factors. The cause factors are of great importance for system safety, and investment in them tends to create compound returns.

### 4.4. Establishment of Reachability Matrix

The reachability matrix K was obtained via the total influence matrix and the threshold value λ. By substituting λ into Equation (9), the weak relationships of the factors were eliminated. The reachability matrix was constructed and is reported in Table S3.

### 4.5. Establishment of Hierarchical Structure Model

Reachable set $R_{i}$, antecedent set $A_{i}$ and collective set $C_{i}$ of each factor were obtained via Equations (10)–(12). The results are represented in Table 5.

In Table 5, factors S_3_, S_4_, S_10_ and S_11_, Ci=Ri (*i* = 3, 4, 10 and 11), S_3_, S_4_, S_10_, and S_11_ were classified into L_1_. The *i*-th (*i* = 3, 4, 10, and 11) rows and columns were deleted to create a new reachability matrix. For factors S_15_ and S_16_ in this new matrix, Ci=Ri (*i* = 15 and 16), then S_15_ and S_16_ were classified into L_2_. This step was repeated until all influencing factors in the EPEM system were classified into 5 layers: L_1_, L_2_, L_3_, L_4_, and L_5_. L_1_ denotes the direct cause layer with four factors such as outfits and belongings S_3_, behavior S_4_, operating status S_10_ and safety devices S_11_. Signage S_15_ and physical environment S_16_ are in the indirect cause layer L_2_. L_3_ and L_4_ are the transition cause layers. L_3_ has eight influencing factors including age and gender S_1_, mental or physical condition S_2_, safety cognition S_7_, safety skill S_8_, maintenance S_12_, installation location S_13_, size of space S_14_ and emergency plan S_21_. L_4_ consists of three factors including knowledge about escalators S_5_, safety awareness S_6_ and information exchange S_19_. L_5_ is the root cause layer, including five factors that are settings and components S_9_, safety rules S_17_, safety supervision S_18_, safety culture S_20_, and safety education S_22_.

**Figure 3:**
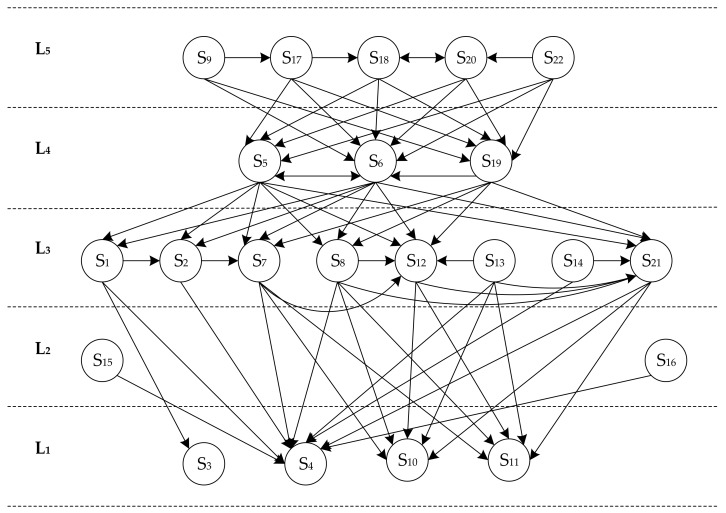
The hierarchical structure model of the influencing factors.

Finally, a hierarchical structure model was established and is illustrated in Figure 3, which clearly reflects the hierarchical structure of the influencing factors and the related influencing mechanisms.

(1) Management priority and preventive measures should be arranged according to the hierarchy level. The rule is that the higher the hierarchy level, the more attention deserved. Root cause factors in L_5_ are the most fundamental and important factors in the EPEM system. Accordingly, optimization of escalator settings and components, strengthening of safety supervision, and implementation of safety education must be emphasized as the top priority for creating long-term system safety. Transition factors in L_4_ and L_3_ connect the root cause layer and the direct cause layer in the system. Enhancing these factors helps to form a positive loop for higher safety levels. Outfits and belongings S_3_, behavior S_4_, operating status S_10_ and safety devices S_11_ are the direct causes of EIs. These factors should be closely monitored and promptly identified with effective and timely responses so as to prevent the occurrence of EIs.

(2) Factors influencing system safety are interactive, and the interaction should be considered when implementing preventive measures. Factors directly or indirectly influence the factors in lower levels. Factors in the same level also influence each other. In particular, four environmental factors in L_2_ and L_3_ include installation location S_13_, size of space S_14_, signage S_15_, and physical environment S_16_ influence the factors in L_1_ but are less influenced by other factors. Special attentions should be paid to these factors, and targeted measures should be conducted to mitigate the environment risk.

(3) Factors belonging to the same category are distributed on the same or adjacent hierarchies. Four of management factors include safety rules S_17_, safety supervision S_18_, safety culture S_20_, and safety education S_22_ are in L_5_, and information exchange S_19_ and emergency plan S_21_ are in L_4_ and L_3,_ respectively. Passenger characteristic factors are distributed in L_4_ and L_3_, and passenger behavior factors in L_1_. Environmental factors are located in L_3_ and L_2_. The exception is escalator factors. One is distributed in L_4_, two in L_3_, and two in L_1_. A general rule is that management factors affect passenger characteristic factors and environmental factors, and characteristic factors and environmental factors affect passenger behavior factors and escalator factors.

## 5. Discussion

### 5.1. Academic Implications

The hierarchical structure of factors influencing EIs were deduced using the proposed method. With DEMATEL, influencing factors were divided into cause factors and effect factors, and the importance of all factors is identified. With ISM, the nonlinear and complicated EPEN system was decomposed into five hierarchies according to the relationships of influencing factors.

The distinguishing features of the ISM-DEMATEL method are that it is systematic and quantitative. First, the method stems from system theory, and the analysis is summarized in a hierarchical structure model. The five hierarchies of factors provide a holistic scenario for understanding the EPEN system. Second, the analysis is based solely on mathematical equations.

Additionally, the influencing factors in this paper were determined based on both news reports cases and related literatures. The combination of two data sources to identify factors is more objective and authentic than reliance on single data source.

### 5.2. Managerial Implications

The hierarchical structure provides a visualization of interrelationships and interdependences among the influencing factors of EIs. It can serve as a useful reference for escalator incident prevention.

The corresponding five-layer countermeasures are proposed to reduce escalator-related injuries.

(1) The top layer measures are to implement safety education and to create a safety climate. A prime example is a more-than-ten-year-old education program called the “Safe-T rider program” in the U.S. that educates school children about how to behave on elevators and escalators. Government management departments of safety in China can consider conducting a similar national escalator safety education program utilizing multi-media and field education modules. Additionally, the public can be educated through publicity campaigns and educational videos. Educational videos and public service announcements on escalator safety can be filmed and broadcasted on mainstream media and social media such as TV channels and Weibo. These efforts should target children and elders. Escalator safety knowledge should be compiled into the safety education textbook for primary students. For elders, watching safety education performances organized by communities is a relaxing and effective way to learn about escalator-related knowledge.

(2) The fourth layer measures are standardizing escalator safety rules and strengthening safety supervision. New standard rules of riding escalators should be formulated and implemented. Escalator safety rule-makers should consist of professionals with escalator safety knowledge and practice, such as escalator technicians, safety managers, safety experts and academics. The formulation of safety rules should refer to the internationally established safety rules for escalators and, more importantly, should be based on the characteristics of passengers and incidents found in safety management practice in China. For example, the rule of walking on the left and standing to the right that originated in the U.K. has been accepted and practiced by escalator passengers worldwide. However, this rule has recently been banned in some cities in China, like Shanghai, Nanjing and Guangzhou, as the practice proven to be unsafe for passengers and damaging to the escalator. Under these circumstances, prohibition of walking on escalators should be written into the safety rules and implemented nationwide. Ensure passengers understand and follow safety rules through communication and supervision.

(3) The third layer measures are to optimize escalator settings and components and to strengthen maintenance. For escalator manufacturers, the construction and installation of escalators must strictly accord with the relevant international standards. The investment in research and development of safety devices should be encouraged since escalator safety could be much improved through the use of high-quality safety devices and equipment. Narrowing the gap between the step and balustrade skirt is another improvement worthy of investment that can reduce the occurrence of the common injury of entrapment. For escalator owners, maintenance on escalators should occur frequently. Quality escalator combined with a robust maintenance program should enable the equipment to last for thirty years. Reliable maintenance helps to reduce overall operating costs and reduce the risk to escalator passengers.

(4) The second layer measures focus on enhancing the management of the environment around escalators. When installing escalators, the specific location of the escalator should be optimized to provide a comfortable and safe environment for passengers. Wet floors and obstacles should be cleaned up in a timely manner. Signage with safety rules should be placed at the entrance to every escalator. Broadcasting escalator safety messages can alert passengers and enhance their safety.

(5) The first layer measures focus on correcting passenger behavior. Passengers are supposed to actively participate in safety education program, learn more escalator safety knowledge and behave properly on escalators. Some of the escalator injuries, such as stumbles and entrapments, are age-specific. Hence, the preventive measures should also be age-specific. Young children should be supervised by parents while riding escalators. They also need to be informed that escalators are dangerous and are not places for jumping or playing. Elders, unhealthy passengers and passengers carrying large luggage or strollers should take elevators instead of escalators. Passengers who wear high-heel shoes, Crocs, long scarves, and long shirts should be on high alert to avoid being entrapped.

## 6. Conclusions

This study revealed the five-hierarchy structure of factors influencing EIs in China. Importantly, root cause factors, including settings and components, safety rules, safety supervision, safety culture, and safety education, were identified and should be given priority when taking preventive measures. The hierarchical structure model suggests that occurrence of EIs can be reduced in the short-term by correcting passenger behavior and in the long-term by enhancing safety education. The proposed five-layer preventive measures provide both theoretical and practical support for managing the safety of escalator passengers. The findings contribute to passenger safety research by offering a deeper understanding of interactive influencing mechanisms of the EPEM system.

## Figures and Tables

**Table 1:** Part of serious escalator-related incidents (EIs) in China.

Time	Place	Cause	Consequence
4 March 2008	Subway station, Beijing	Abnormal noise causing panic	13 injured
24 March 2009	Subway station, Beijing	Escalator reverse malfunction	4 injured (Elders)
14 December 2010	Subway station, Shenzhen	Escalator reverse malfunction	25 injured
5 July 2011	Subway station, Beijing	Escalator reverse malfunction	30 injured (1 dead)
10 July 2011	Subway station, Shenzhen	Escalator reverse malfunction	4 injured
29 March 2013	Aquarium, Xi’an	Escalator sudden deceleration	19 injured (Children)
18 April 2013	Shopping mall, Shenzhen	Bending over to tie shoes at exit	10 injured (Children)
2 April 2014	Subway station, Shanghai	Escalator reverse malfunction	13 injured
18 June 2014	Subway station, Shanghai	Baby stroller is stuck in escalator	10 injured
10 November 2015	Shopping mall, Nanjing	Passengers congest at exit	16 injured (Children)
18 February 2016	Subway station, Ningbo	Escalator reverse malfunction	5 injured
17 October 2016	Train station, Hangzhou	Passenger falls, causing congestion	9 injured (Elders)
25 March 2017	Shopping mall, Hong Kong	Escalator sudden stop	17 injured

Notes: Statistics collected from China’s mainstream media.

**Table 2:** High-frequency keywords of EIs news reports.

Keywords	Freq.	Keywords	Freq.	Keywords	Freq.
Escalator	417	Women	54	Slip	24
Child	249	Supermarket	47	Draw	19
Stumble	229	stuck	45	Arm	17
Elder	161	Hand	41	Play	17
Injure	130	Roll	40	Fracture	15
Medical care	90	Plaza	35	Luggage	13
Subway station	79	Foot	35	Fall	11
Handrail	68	Gap	35	Head	11
Entrap	67	Emergency button	33	Death	11
Shopping mall	62	Finger	27	Graze	11

**Table 3:** Influencing factors of EIs.

Symbol	Factor	Meaning/Explanation	Reference
S_1_	Age and gender	Passengers’ age and gender directly affect their perception, judgment and behavior which are closely related to the occurrence of EIs. Children and elders are the most common victims of EIs in China.	Xing et al., 2017; O’Neil et al., 2008; Chi et al., 2006; Schminke et al., 2013 [1,2,3,6].
S_2_	Mental or physical condition	Mental or physical condition affects passengers’ cognition, decision-making and action ability. Drunkenness, unhealthiness and carelessness are major causes of EIs.	Xing et al., 2017; Chi et al., 2006; Schminke et al., 2013; Basir et al., 2017 [1,3,6,23].
S_3_	Outfits and belongings	Improper outfits and large belongings are likely to result in EIs, which include Crocs shoes, high-heeled shoes, scarves, loose shoelaces, drawstrings, mittens, huge luggage, shopping carts, and strollers.	Xing et al., 2017; Basir et al., 2017 [1,23].
S_4_	Behavior	Behavior refers to passengers’ actions while riding an escalator. Multi-tasking, not holding the handrail, stepping on step edges, running on escalators and playing on escalators are common improper behaviors which lead to EIs.	Xing et al., 2017; Chi et al., 2006; Basir et al., 2017 [1,3,23].
S_5_	Knowledge about escalators	Knowledge about escalators reflects passengers’ comfort and common sense on escalator such as familiarity with escalator structure, knowing the place of emergency buttons and understanding the safety rules of riding escalators.	Alonso et al., 2018; Michael, 2005 [35,36].
S_6_	Safety awareness	Safety awareness refers to a sense for the external conditions that may pose a threat to passenger safety. A high-level of safety awareness helps passengers follow the safety rules and behave properly when riding an escalator	Alonso et al., 2018; Al-sharif, L. 2005 [35,37].
S_7_	Safety cognition	Safety cognition is the ability to evaluate the existing state of safety. The perception of danger is the precondition for making response decisions.	Alonso et al., 2018; Michael, 2005 [35,36].
S_8_	Safety skill	Safety skill refers to the passengers’ knowledge of escalator safety and ability to handle emergency situation. Passengers with safety skills can confidently handle various situation when riding an escalator, reducing the consequences of EIs.	Michael, 2005; Al-sharif, L. 2005 [36,37].
S_9_	Settings and components	Settings and components of escalators include length, width, slope, operating speed, chains, steps, shirts, handrails and electric equipment. These factors directly affect the probability of EIs.	Xing et al., 2017; Li et al. 2014; Isnaini Janipha et al., 2018; Bardyshev et al., 2017; Lai et al.,2011; [1,10,38,39,40].
S_10_	Operating status	A strong operating status can greatly reduce the probability of EIs. Malfunctions such as reverse malfunction and sudden stops are the main causes of EIs in China.	Basir et al., 2018; Al-sharif, L. 2005; Bardyshev et al., 2017; Li et al., 2015 [23,37,39,41].
S_11_	Safety devices	Safety devices include comb plates (to prevent passengers from tripping), balustrade skirts (to prevent clothes from getting stuck), automatic service break (to enable the escalator to stop smoothly if the drive chain or step chain is broken or if an object gets stuck into the handrail’s inlet) and emergency buttons.	Al-sharif, L. 2012; Isnaini Janipha et al. 2018; Kuutti et al., 2013 [30,38,42].
S_12_	Maintenance	The maintenance aspect covers engineering areas such as the periodic preventative maintenance and remedial works as well as items such as cleaning (to prevent accumulation of dust and dirt that can lead to escalator fires).	Al-Sharif et al. 2012; Bardyshev et al., 2017; Kuutti et al., 2013 [30,39,42].
S_13_	Installation location	Installation location means the specific place in which the escalator is installed. The location factor covers venue (subway station, train station and shopping mall), floor, and distances to stairs, elevators and exits.	Bardyshev et al., 2017; Ma et al., 2009; [39,43].
S_14_	Size of space	Size of space includes landing platforms and overhead room. Enough room for passengers can reduce the risk of congestion and stampede when they approach or leave an escalator.	Li et al. 2014; Lai et al.,2011; Ma et al., 2009; Dolan et al., 2006 [10,41,43,44]
S_15_	Signage	Signage includes path signage, commercial advertising and any other signage that guides or distracts passengers. Signage around the escalator has a positive as well as negative impact on passenger safety.	Al-sharif, L. 2005; Dolan et al., 2006 [37,44].
S_16_	Physical environment	Physical environment denotes the environmental factor that can affect system safety and mainly includes lighting, noise, temperature, humidity, dusts and hazardous gas. Environmental changes affect passengers’ physical and psychological state, and an undesirable physical environment may induce unsafe behavior.	Li et al., 2015; Ma et al., 2009; [41,43].
S_17_	Safety rules	Safety rules refer to the safety-related regulations and constraints on the escalator passenger, instructing passengers on what they should do and what they should not do when riding escalators.	Schminke et al., 2013; Shi et al., 2012 [6,45].
S_18_	Safety supervision	Safety supervision means on-site management of escalators and passengers by safety staff and managers. Supervision helps to correct improper behavior and identify possible safety risks.	Schminke et al., 2013; Shi et al., 2012 [6,45].
S_19_	Information exchange	Information exchange denotes the exchange of safety information and safety rules between passengers and safety staff. The form of communication includes face-to-face conversation, radio, video, and signage.	Al-sharif, L. 2007; Shi et al., 2012 [37,45].
S_20_	Safety culture	Safety culture refers to the ideas and feelings about safety, including safety philosophy, norms of safety behavior, and safety attitude. A strong safety culture is critical to public transportation safety.	Alonso et al., 2018; Michael, 2005 [35,36].
S_21_	Emergency plan	Emergency plan is a pre-defined action plan for emergency actions that occurs in response to possible incidents.	Kauffmann and Kikuchi, 2013; Shi et al., 2012; Kadokura et al., 2012 [11,45,46].
S_22_	Safety education	Safety education denotes the training and education for the public on safety cognition, safety knowledge, and safety skill.	Alonso et al., 2018; Al-sharif, L. 2005 [35,37,47].

**Table 4:** Results of influencing and influenced degrees, centrality and causality.

	S_1_	S_2_	S_3_	S_4_	S_5_	S_6_	S_7_	S_8_	S_9_	S_10_	S_11_	S_12_	S_13_	S_14_	S_15_	S_16_	S_17_	S_18_	S_19_	S_20_	S_21_	S_22_
*f_i_*	0.67	0.59	0.29	0.27	1.09	1.12	0.73	0.86	1.47	0.30	0.49	0.78	0.87	0.80	0.32	0.49	1.60	1.46	1.11	1.27	0.99	1.86
*e_i_*	0.88	0.81	1.03	2.17	0.73	1.01	1.27	0.89	0.06	1.19	1.21	1.30	0.39	0.33	0.61	0.13	0.79	0.90	1.09	0.88	1.44	0.29
*m_i_*	1.54	1.40	1.32	2.43	1.81	2.14	2.00	1.75	1.53	1.49	1.70	2.08	1.26	1.13	0.93	0.62	2.38	2.36	2.21	2.15	2.43	2.14
*n_i_*	−0.21	−0.23	−0.74	−1.90	0.36	0.11	−0.54	−0.03	1.42	−0.89	−0.73	−0.52	0.48	0.47	−0.29	0.36	0.81	0.55	0.02	0.39	−0.45	1.57

**Table 5:** The reachable set, antecedent set and collective set of factors.

Factor	Reachable Set	Antecedent Set	Collective Set	Layers
S_1_	S_1_–S_4_	S_1_, S_5_, S_6_, S_9_, S_17_, S_18_, S_20_, S_22_	S_1_	L_3_
S_2_	S_2_, S_4_, S_7_	S_1_, S_2_, S_5_, S_6_, S_17_, S_18_, S_20_, S_22_	S_2_	L_3_
S_3_	S_3_	S_1_, S_3_, S_5_, S_6_, S_17_−S_20_, S_22_	S_3_	L_1_
S_4_	S_4_	S_1_−S_9_, S_11_, S_13_−S_22_	S_4_	L_1_
S_5_	S_1_–S_8_, S_10_–S_12_, S_19_, S_21_	S_5_, S_6_, S_17_, S_18_, S_20_, S_22_	S_5_, S_6_	L_4_
S_6_	S_1_–S_8_, S_10_–S_12_, S_21_	S_5_, S_6_, S_9_, S_17_−S_20_, S_22_	S_5_, S_6_	L_4_
S_7_	S_4_, S_7_, S_10_–S_12_	S_2_, S_5_−S_7_, S_9_, S_13_, S_17_−S_20_, S_22_	S_7_	L_3_
S_8_	S_4_, S_8_, S_10_−S_12_, S_21_	S_5_, S_6_, S_8_, S_17_−S_20_, S_22_	S_8_	L_3_
S_9_	S_1_, S_4_, S_6_, S_7_, S_9_, S_10_−S_15_, S_17_−S_21_	S_9_	S_9_	L_5_
S_10_	S_10_	S_5_−S_10_, S_12_, S_13_, S_17_−S_21_	S_10_	L_1_
S_11_	S_11_	S_5_−S_9_, S_11_−S_13_, S_17_−S_22_	S_11_	L_1_
S_12_	S_10_, S_11_, S_12_, S_21_	S_5_−S_9_, S_12_, S_13_, S_17_, S_18_−S_22_	S_12_, S_21_	L_3_
S_13_	S_4_, S_7_, S_10_−S_13_, S_21_	S_9_, S_13_, S_21_	S_13_, S_21_	L_3_
S_14_	S_4_, S_14_, S_21_	S_9_, S_14_	S_14_	L_3_
S_15_	S_4_, S_15_	S_9_, S_15_	S_15_	L_2_
S_16_	S_4_, S_16_	S_16_	S_16_	L_2_
S_17_	S_1_−S_8_, S_10_−S_12_, S_17_−S_21_	S_9_, S_17_, S_20_	S_17_, S_20_	L_5_
S_18_	S_1_−S_8_, S_10−_S_12_, S_18_−S_21_	S_9_, S_17_, S_18_, S_20_, S_22_	S_18_, S_20_	L_5_
S_19_	S_3_−S_8_, S_10_−S_12_, S_19_, S_21_	S_5_, S_9_, S_17_−S_20_, S_22_	S_19_	L_4_
S_20_	S_1_−S_8_, S_10_−S_12_, S_17_−S_21_	S_9_, S_17_, S_18_, S_20_, S_22_	S_17_, S_18_, S_20_, S_22_	L_5_
S_21_	S_4_, S_10_−S_13_, S_21_	S_5_, S_6_, S_8_, S_9_, S_12_−S_14_, S_17_ −S_22_	S_13_, S_21_	L_3_
S_22_	S_1_−S_8_, S_11_, S_12_, S_18_−S_22_	S_22_	S_22_	L_5_

## Supplementary Materials

The following are available online at https://www.mdpi.com/1660-4601/16/14/2478/s1.
