# Supplementary figures and images for: Factors Influencing Escalator-Related Incidents in China: A Systematic Analysis Using ISM-DEMATEL Method
Source: Int J Environ Res Public Health. 2019 Jul 11;16(14):2478. doi: 10.3390/ijerph16142478 (PMC6678537; doi:10.3390/ijerph16142478)

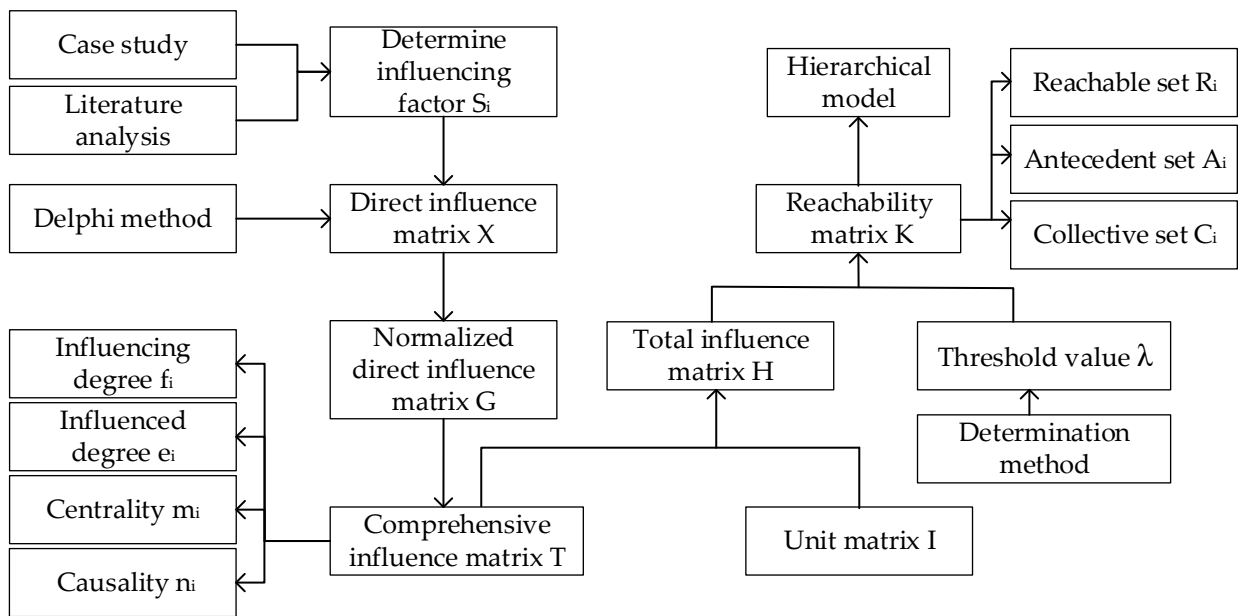

Supplement: Supplementary file 1 [file ijerph-16-02478-s001.zip › Figures and data/Figure 1.pdf]

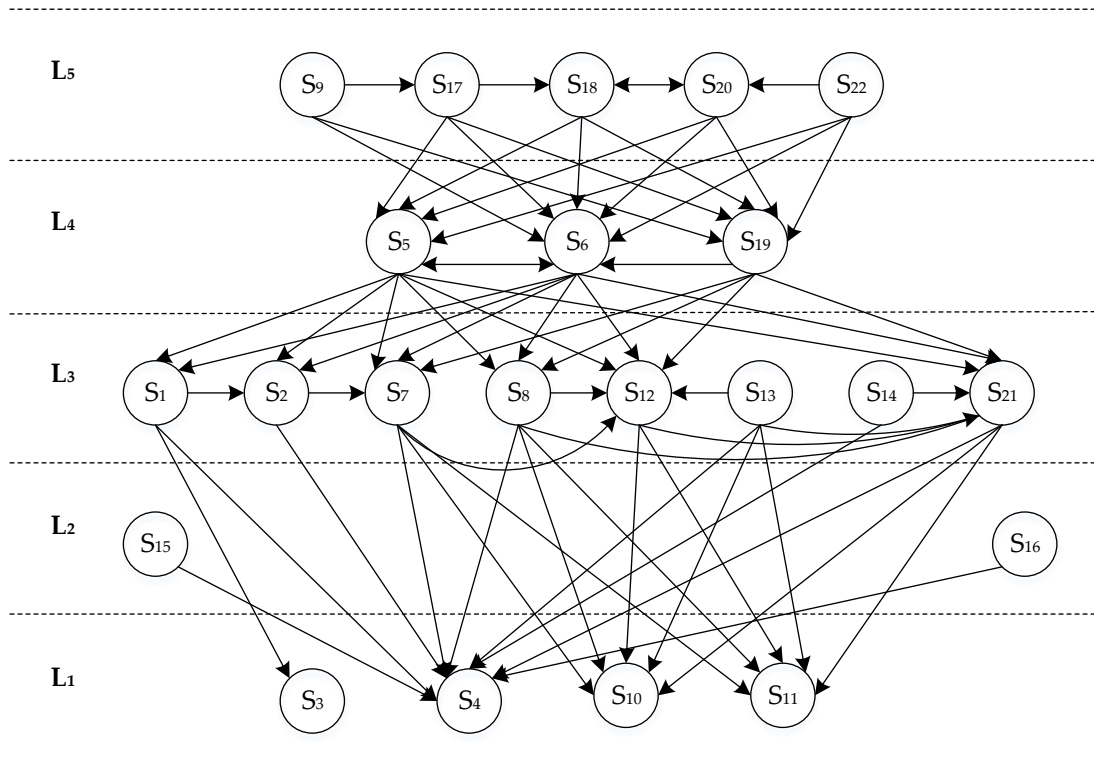

Supplement: Supplementary file 1 [file ijerph-16-02478-s001.zip › Figures and data/Figure 3.pdf]
